# Interleukin-37 Ameliorates Influenza Pneumonia by Attenuating Macrophage Cytokine Production in a MAPK-Dependent Manner

**DOI:** 10.3389/fmicb.2019.02482

**Published:** 2019-10-30

**Authors:** Feifei Qi, Mingya Liu, Fengdi Li, Qi Lv, Guanpeng Wang, Shuran Gong, Shunyi Wang, Yanfeng Xu, Linlin Bao, Chuan Qin

**Affiliations:** ^1^NHC Key Laboratory of Human Disease Comparative Medicine, The Institute of Laboratory Animal Sciences, Peking Union Medical College Hospital (CAMS), Beijing, China; ^2^Beijing Key Laboratory for Animal Models of Emerging and Reemerging Infectious, The Institute of Laboratory Animal Sciences, Peking Union Medical College Hospital (CAMS), Beijing, China

**Keywords:** A/California/07/2009 (H1N1), Interleukin-37, viral pneumonia, macrophages, inflammation

## Abstract

Viral pneumonitis caused by influenza A (H1N1) virus leads to high levels of morbidity and mortality. Given the limited treatment options for severe influenza pneumonia, it is necessary to explore effective amelioration approaches. Interleukin-37 (IL-37) has been reported to inhibit excessive immune responses and protect against a variety of inflammatory diseases. In this study, by using BALB/c mice intranasally infected with A/California/07/2009 (H1N1), we found that IL-37 treatment increases the survival rate and body weight, and reduces the pulmonary index, impaired the lung injury and decreased production of pro-inflammatory cytokines in the BALF and lung tissue. Moreover, IL-37 administration enhanced not only the percentage of macrophages, but also the percentage of IL-18Rα^+^ macrophages, suggesting that enhancing the macrophages function may improve outcomes in a murine model of H1N1 infection. Indeed, macrophages depletion reduced the protective effect of IL-37 during H1N1 infection. Furthermore, IL-37 administration inhibited MAPK signaling in RAW264.7 cells infected with H1N1. This study demonstrates that IL-37 treatment can ameliorate influenza pneumonia by attenuating cytokine production, especially by macrophages. Thus, IL-37 might serve as a promising new target for the treatment of influenza A-induced pneumonia.

## Introduction

Influenza H1N1 infection induced “flu”-like illness or pneumonia depends on the infecting strain, host immune system, and environmental factors (Rice et al., 2012; Daoud et al., 2019). H1N1 pneumonia may progress rapidly, resulting in severe respiratory distress syndrome or refractory hypoxemia, is associated with a longer hospital stay with higher mortality compared to bacterial pneumonia (Perez-Padilla et al., 2009; Rello et al., 2009; Hermann et al., 2017). Thus, H1N1 infection is considered a more life-threatening disease (Ramsey and Kumar, 2013; Liu et al., 2016).

Increasing evidence has demonstrated that it is an excessively activated immune response, not a direct viral infection that leads to the increasing influenza pneumonia severity (Morita et al., 2013; Uematsu et al., 2015). Although various prevention and treatment methods have been used for viral diseases, the limited treatment options for severe influenza pneumonia prioritize the need for the discovery of effective therapies.

Interleukin-37 (IL-37), a novel member of the IL-1 family, inhibits systemic and local inflammation by reducing the levels of pro-inflammatory mediators (Nold et al., 2010; Boraschi et al., 2011; Al-Anazi et al., 2019). IL-37 binds to the IL-18Rα chain, and then recruits TIR-8/IL-1R8/SIGIRR to execute its anti-inflammatory effects (Kumar et al., 2002; Boraschi and Tagliabue, 2013; Dinarello and Bufler, 2013; Lunding et al., 2015; Nold-Petry et al., 2015). IL-37 has been shown to increase the survival rate and body weight, and downregulated the production of IL-6 and IL-17A in a coxsackievirus B3-induced model of murine viral myocarditis (An et al., 2017). In addition, comparing with wild-type (WT) mice, a low dose of mouse-adapted H1N1-induced morbidity and the decreases in body weight are significantly attenuated in IL-37tg mice, which express human IL-37 isoform b precursor transgene (Davis et al., 2017). Moreover, IL-37 significantly attenuates pulmonary eosinophilia, CCL11 production and airway hyper-reactivity in a murine asthma model (Lv et al., 2018). Increasing evidence suggests that IL-37 can inhibit excessive immune responses and protect against a variety of inflammatory diseases, autoimmune diseases, and tumors. However, little is known about the function of IL-37 in the influenza-infected murine model, particularly the regulatory role in viral pneumonia induced by A/California/07/2009 (H1N1) infection. Thus, in the present study, we focused on IL-37 treatment in H1N1-infected mice, to investigate the therapeutic effect and the mechanisms by which IL-37 treatment ameliorates influenza pneumonia.

## Materials and Methods

### Animals and Viruses

Specific pathogen-free, 4- to 6-week-old female BALB/c mice were obtained from Vital River Laboratories (Beijing, China). The seasonal influenza A virus strain A/California/07/2009 (H1N1) was provided by the Institute of Laboratory Animal Science, Peking Union Medical College, China. All experiments were performed in biosafety level 2 facilities in compliance with governmental and institutional guidelines. The experimental protocol was evaluated and approved by the Institute of Animal Use and Care Committee of the Institute of Laboratory Animal Science, Peking Union Medical College (BLL19004).

### Therapeutic Treatments

Oseltamivir phosphate capsules were purchased from Roche Pharmaceutical Co., Ltd. (Shanghai, China). The pGEM-T-IL-37b plasmid was kindly supplied by Dr. RF. Wei., Institute of Laboratory Animal Science, Peking Union Medical College, China. Individual mice were anesthetized with tribromoethanol and inoculated intranasally with 50 μl (10^4.3^ TCID_50_) of allantoic fluid containing influenza A/California/07/2009 (H1N1) virus. Subsequently, the mice were chronically intragastrically administered oseltamivir phosphate (30 mg/kg) for 5 days, and the animals were inoculated with IL-37 (12.5 μg/kg) *via* intravenous or intranasal administration at three separate time points (2, 24, and 48 h post infection). Seven mice were selected randomly from each group for monitoring the disease signs, weight loss, and mortality daily up to 14 days post inoculation (d.p.i.). The remaining mice in each group were euthanized at 6 d.p.i. and blood samples, bronchoalveolar lavage fluid (BALF), and lung tissues were collected for the assessment of lung histology, pro-inflammatory cytokines, and immune cell counts.

### Preparation of Single Cell Suspensions From the Lung

Mice were anesthetized and the lung was flushed *in situ* with 20 ml of phosphate-buffered saline (PBS) *via* cannulation of the heart to remove the intravascular blood pool. Minced lung tissues were incubated at 37°C for 1 h on a rocker with 200 μg/ml collagenase D and 40 μg/ml DNase I (Roche Molecular Biochemicals) in 10 ml of DMEM supplemented with 10% FBS. Single cell suspensions from the digested lung were filtered through a 75-μm strainer and then collected through density-gradient centrifugation with lymphocyte separation solution. The immune cells were washed twice with Hank’s solution and suspended in Hank’s solution.

### Flow Cytometry Analysis

Cells were pre-incubated with Zombie Aqua^™^ Fixable Viability Kit (Biolegend) and purified rat anti-mouse CD16/CD32 (Mouse BD Fc Block^™^, 2.4G2, BD Pharmingen^™^) ice for 15 min at room temperature. For the extracellular cell marker analysis, the cells were incubated with the following fluorescein-conjugated antibodies for 30 min: BV421-anti-CD11b (M1/70, Biolegend), FITC-anti-CD45 (30-F11, Biolegend), BV605-anti-F4/80 (BM8, Biolegend), PE-anti-IL-18Rα (Miltenyi Biotec), AF647-anti-SIGIRR (Santa Cruz), BV421-anti-CD3ε (145-2C11, Biolegend), PE-Cy7-anti-CD4 (RM4-5, BD Biosciences), and PerCP-Cy5.5-anti-CD8a (53-6.7, BD Biosciences). Finally, samples were acquired using a fluorescence-activated cell sorting (FACS) Aria II system (BD Biosciences). The data were analyzed using a Kaluza analysis and FlowJo 10.1 software.

### Preparation of Lung Homogenate Supernatant

Lung homogenates were prepared by homogenizing perfused whole lung tissue using an electric homogenizer for 2 min 30 s in 1 ml of PBS. The homogenates were centrifuged at 3,000 rpm for 10 min at 4°C. The supernatant was collected and stored at −80°C.

### Analysis of Bronchoalveolar Lavage Fluid

Bronchoalveolar lavage fluid (BALF) was collected by washing the lungs of sacrificed mice twice with 1 ml PBS. The PBS was then recovered after 1 min and centrifuged at 1,500 rpm for 10 min at 4°C. The supernatant was collected and stored at −80°C. Total cellular infiltration in the BALF was assessed using a hemocytometer; cytosine slides were fixed and stained with Wright-Giemsa stain, and the composition was assessed in a blinded manner by counting 200 or more cells using a light microscope.

### Clodronate Treatment

To deplete macrophages, ready-made clodronate liposomes and control liposomes (FormuMax; CA, USA) were intranasally administered to mice using the manufacturer’s recommending dose 1 day before and 1 day after A/California/07/2009 (H1N1) infection. Mice were monitored for signs of disease, weight loss, and mortality upto 14 d.p.i.

### Quantification of Cytokines

The concentrations of granulocyte colony-stimulating factor (G-CSF), granulocyte-macrophage colony-stimulating factor (GM-CSF), interferon-γ (IFN-γ), monokine induced by IFN-γ (MIG), interleukin (IL)-1α, IL-1β, IL-4, IL-5, IL-6, IL-10, IL-12/IL-23p40, IL-13, IL-17A, RANTES, monocyte chemoattractant protein 1 (MCP-1), macrophage inflammatory protein 1α (MIP-1α), MIP-1β, and tumor necrosis factor (TNF) in the serum and BALF samples were determined by flow cytometry (FACS Aria II, BD, USA) using the Cytometric Beads Array (CBA) Kits, according to the manufacturers’ instructions (BD, USA). Briefly, 50 μl of each testing sample were labeled in duplicate with equal volumes of diluted FlexSet capture beads at room temperature for 1 h and treated with PE-conjugated detection reagent. After washing, the captured cytokines and chemokines were analyzed by flow cytometry.

### Hematoxylin and Eosin Staining

For each mouse, the whole right lung was fixed in 10% formalin for 24 h and then embedded in paraffin for histological examination. The lung tissue sections (4 μm) were deparaffinized and hydrated using xylene and an alcohol gradient and then, stained with Hematoxylin and Eosin (H&E). The histopathology of the lung tissue was observed by light microscopy.

### Quantitative Real-Time Polymerase Chain Reaction Analysis

Total RNA was isolated from individual samples using an RNeasy Mini kit, according to the manufacturer’s instructions (Qiagen, Hilden, Germany). The RNA was reversely transcribed into cDNA using random primers and a SuperScript II reverse transcriptase reaction mixture (Invitrogen). The target gene mRNA transcripts were determined by RT-PCR using SYBR Green PCR Master Mix, specific primers, and a 7500 PCR system (ABI, USA). Primer sets for individual genes are shown in Table 1.

**Table 1:** Primer sequences used for RT-PCR analysis.

Gene	Forward (5′-3′)	Reverse (5′-3′)
β-actin	CAACGAGCGGTTCCGATG	GCCACAGGATTCCATACCCA
IL-6	TCTATACCACTTCACAAGTCGGA	GAATTGCCATTGCACAACTCTTT
IL1α	TCTCAGATTCACAACTGTTCGTG	AGAAAATGAGGTCGGTCTCACTA
IL-1β	CAACCAACAAGTGATATTCTCCATG	GATCCACACTCTCCAGCTGCA
RANTES	GCTGCTTTGCCTACCTCTCC	TCGAGTGACAAACACGACTGC
IP10	CCAAGTGCTGCCGTCATTTTC	GGCTCGCAGGGATGATTTCAA
MCP1	AGTAGGCTGGAGAGCTACAA	GTATGTCTGGACCCATTCCTTC
MIP1α	TTCTCTGTACCATGACACTCTGC	CGTGGAATCTTCCGGCTGTAG
MIP1β	TTCCTGCTGTTTCTCTTACACCT	CTGTCTGCCTCTTTTGGTCAG
MIG	TCCTTTTGGGCATCATCTTCC	TTTGTAGTGGATCGTGCCTCG
IFN-γ	TATCTGGAGGAACTGGCAAA	GGTGTGATTCAATGACGCTT
IL18Rα	TCACCGATCACAAATTCATGTGG	TGGTGGCTGTTTCATTCCTGT

*The results are normalized to β-actin expression and presented as the fold change in mRNA expression (fold change = 2^−△△CT^)*.

### Cells

Murine macrophage cell lines (RAW264.7) were maintained in Dulbecco’s modified Eagle’s medium (Gibco, Life Technologies, New York) supplemented with 10% FBS, 100 IU/ml penicillin, and 100 μg/ml streptomycin and were incubated at 37°C with 5% CO_2_. RAW264.7 cells in six-well plates were infected with H1N1 at a multiplicity of infection (MOI) of 0.01 for 1 h absorption at 37°C. Then, the cells were washed and cultured with 2 ml of serum-free DMEM containing TPCK-treated trypsin (0.5 mg/ml) antibiotics and 70 μM oseltamivir phosphate with or without IL-37b for 72 h. The cells were collected at 0, 12, and 24 h post infection to detect the expression of cytokines such as IL-6, MCP-1, TNF-α, IL-1β, and IL-1α. The protein levels of MAPKs and NLRP3 were detected by western blot assay at 0, 30, and 60 min after infection.

### Western Blot Assay

The expression of GAPDH, p38, phospho-p38 (p-p38), ERK, phospho-ERK (p-ERK), NF-κB, JNK, phospho-JNK (p-JNK), and NLRP3 (Cell Signaling, Boston, MA, USA) was assessed by western blotting. Protein bands were detected with a chemiluminescent imaging system (Amersham, Freiburg, Germany). The expression of a target protein was normalized to that of GAPDH.

### Statistical Analysis

The data are presented as the mean ± SEM. Analysis of variance (ANOVA) was used to analyze the differences between three or more groups, and *t* tests were used to analyze the differences between two groups. Statistical graphs were obtained using GraphPad Prism 5 software. Differences were considered statistically significant at values of *p* < 0.05.

## Results

### Interleukin-37 Treatment Reduces the Body Weight Recovery Time and Improves the Survival Rate in H1N1-Infected Mice

**Figure 1:**
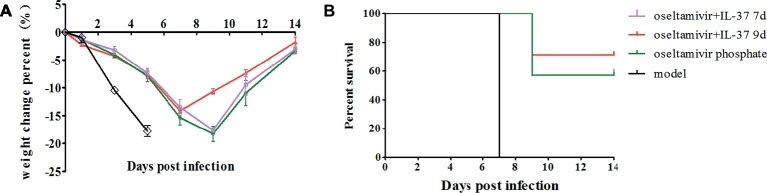
IL-37 treatment reduces the time to body weight recovery onset and decreases mortality in mice during influenza infection. Mice were infected intranasally with 10^4.6^ TCID_50_ of influenza A (H1N1) virus; body weight **(A)** and mortality rates **(B)** were recorded at two different time points of intravenous IL-37 administration. Data are presented as the average values from three independent experiments ± SD (*n* = 7 per group).

To explore the efficacy of the recombinant IL-37 protein, BALB/c mice challenged intranasally with 50 μl H1N1 were treated with oseltamivir phosphate for 5 days with/without recombinant IL-37 for 7 days at 2 h.p.i. As shown in Figure 1A, the body weights of mice treated for 7 days (oseltamivir+IL-37 7d) were not obviously different from those of mice in the oseltamivir phosphate group. Nevertheless, in this study, we extended the IL-37 administration time to 9 days (oseltamivir+IL-37 9d), and the death and body weight changes in the mice (*n* = 7) were monitored for 14 d.p.i. The results showed that the body weights of mice began to increase from 7 d.p.i., which was 2 days earlier than the body weights of mice in the oseltamivir phosphate group began to increase (Figure 1A). A total of 71% of the mice from the oseltamivir+IL-37 9d group survived, whereas the survival rate in the oseltamivir phosphate group was 57% survival. All of the mice in the model group died (Figure 1B), suggesting that IL-37 treatment for 9 days advanced the onset of body weight recovery and improves the survival rate in H1N1-infected mice.

### Intravenous Interleukin-37 Administration in Mice Enhances the Protection Against Influenza Challenge

**Figure 2:**
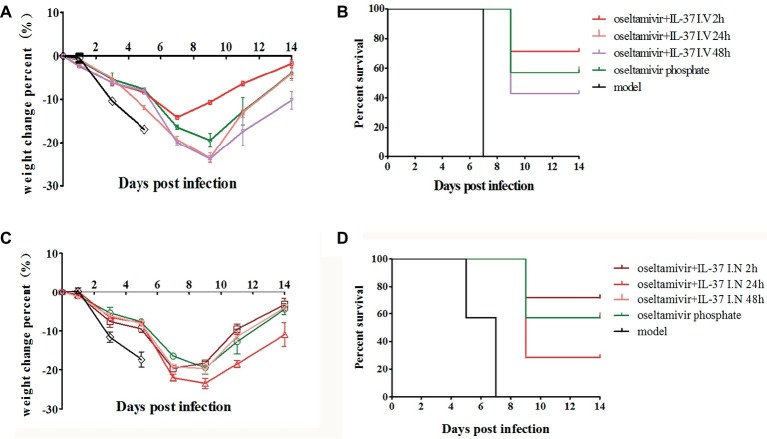
IL-37 intravenous administration offers enhanced protection against influenza challenge in mice. BALB/c mice (*n* = 7 in each group) treated with or without IL-37 were divided into eight groups: the H1N1-infected group (model) and oseltamivir phosphate, intravenous oseltamivir phosphate combined with IL-37 at three separate time points (oseltamivir+IL-37 I. V 2 h, oseltamivir+IL-37 I. V 24 h, oseltamivir+IL-37 I. V 48 h) or intranasal oseltamivir phosphate combined with IL-37 at three separate time points (oseltamivir+IL-37 I. N 2 h, oseltamivir+IL-37 I. N 24 h, oseltamivir+IL-37 I. N 48 h) treatment groups. The body weights **(A,C)** and mortality rates **(B,D)** of mice treated *via* intravenous or intranasal routes were monitored. Data are presented as the average values from two independent experiments ± SD (*n* = 7 per group).

To further explore the best time point and route of administration for IL-37, after infection, the BALB/c mice were divided into eight groups: H1N1 infection (model), oseltamivir phosphate, oseltamivir phosphate combined with intravenous IL-37 administration at three separate time points (oseltamivir+IL-37 I. V 2 h, oseltamivir+IL-37 I. V 24 h, and oseltamivir+IL-37 I. V 48 h) or oseltamivir phosphate combined with intranasal IL-37 administration at three separate time points (oseltamivir+IL-37 I. N 2 h, oseltamivir+IL-37 I. N 24 h, and oseltamivir+IL-37 I. N 48 h). A model group was also administered PBS. Seven mice were selected randomly from each group, and mortality rates and body weight changes in the mice were monitored for 14 d.p.i. Treatment with IL-37 protein in combination with oseltamivir phosphate reduced the time to the onset of body weight recovery, and this reduction varied based on the different administration times. As shown in Figure 2A, the body weights of mice infected with H1N1 decreased and began to increase from the 7th day of IL-37 plus oseltamivir phosphate treatment at 2 h.p.i. (oseltamivir+IL-37 I.V 2 h), whereas the body weights of mice in the oseltamivir phosphate group began to increase on the 9th day; in the model group, the body weight continued to decrease until death. Moreover, animals that received PBS succumbed to infection 5–7 days after viral challenge. When therapeutic treatment was started at 24 h.p.i, 57% of the infected mice survived, which was the same rate as the mice treated with oseltamivir phosphate only. The administration of IL-37 at 2 h.p.i. increased the mouse survival rate to 71%. However, in the group administered IL-37 at 48 h.p.i, the mortality rate increased (Figure 2B).

Similar to the results shown in Figure 2, the survival rate of mice intranasally administered IL-37 at 2 h.p.i., was significantly enhanced compared to that of mice in the oseltamivir phosphate group (Figure 2D). However, the body weight in the intranasal IL-37 treatment group was not significantly increased compared with that in the oseltamivir phosphate group; however, the body weight decrease time was shortened and remained stable at 7–9 d.p.i (Figure 2C). These results demonstrate that intravenous IL-37 administration at 2 h.p.i significantly decreases the mortality of mice infected with H1N1 and shortens the recovery time of infected mice, so intravenous IL-37 administration at 2 h.p.i offers enhanced protection against influenza challenge in mice.

### Interleukin-37 Treatment Reduces Lung Damage in Mice Infected With H1N1 Virus

**Figure 3:**
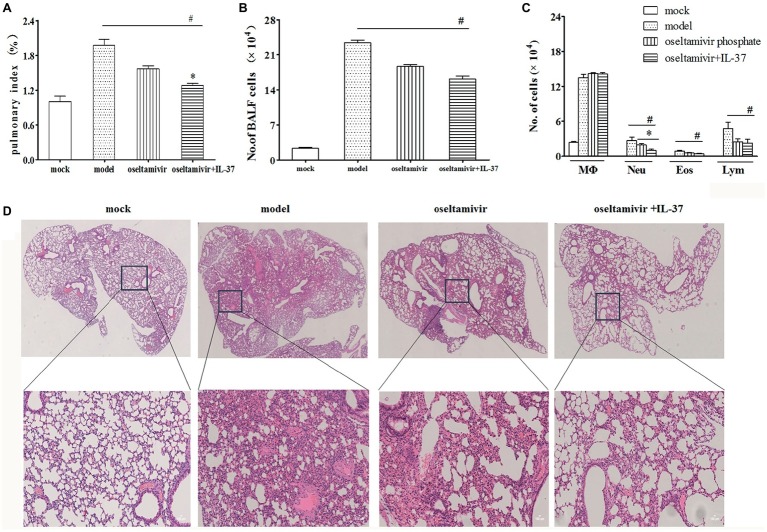
IL-37 treatment attenuates H1N1-induced lung tissue damage *in vivo*. Lungs were obtained from different groups of mice, and the pulmonary index on day 6 post infection was monitored **(A)**. The BALF was harvested on day 6 d.p.i, the number of total BALF cells **(B)** and the proportions of different leukocyte subtypes in the BALF **(C)** were calculated. **(D)** Mouse lung tissues were stained with H&E. Data are representative of three independent experiments with three mice per group (100× magnification). Data are representative of three independent experiments with three mice for each group. *Significant difference (*p* < 0.05), compared with oseltamivir-inoculated mice. ^#^Significant difference (*p* < 0.05), compared with H1N1-infected mice.

In addition, to further validate the therapeutic effect of IL-37, lung tissue was collected to monitor the pulmonary indexes and lung histology. As expected, the IL-37 combined with oseltamivir phosphate administration group had significantly lower pulmonary index values than the other groups (Figure 3A). The BAL fluid was gathered 6 days after infection or IL-37 treatment. The total cell number in the BALF was increased significantly after infection (Figure 3B). However, IL-37 treatment evidently diminished H1N1-induced neutrophilic and eosinophilic airway inflammation (Figure 3C). Additionally, H&E staining of the lung tissue samples showed that the lungs in the model group exhibited many merged, inflated, or enlarged alveoli as well as an increase in the exudation of inflammatory proteins in the alveolar spaces at 6 d.p.i., which was largely decreased in the IL-37-treated group (Figure 3D). These results further indicate that IL-37 could be a useful therapeutic agent in mice with H1N1 infection.

### Interleukin-37 Inhibits the Production of Inflammatory Cytokines in H1N1-Infected Mice

**Figure 4:**
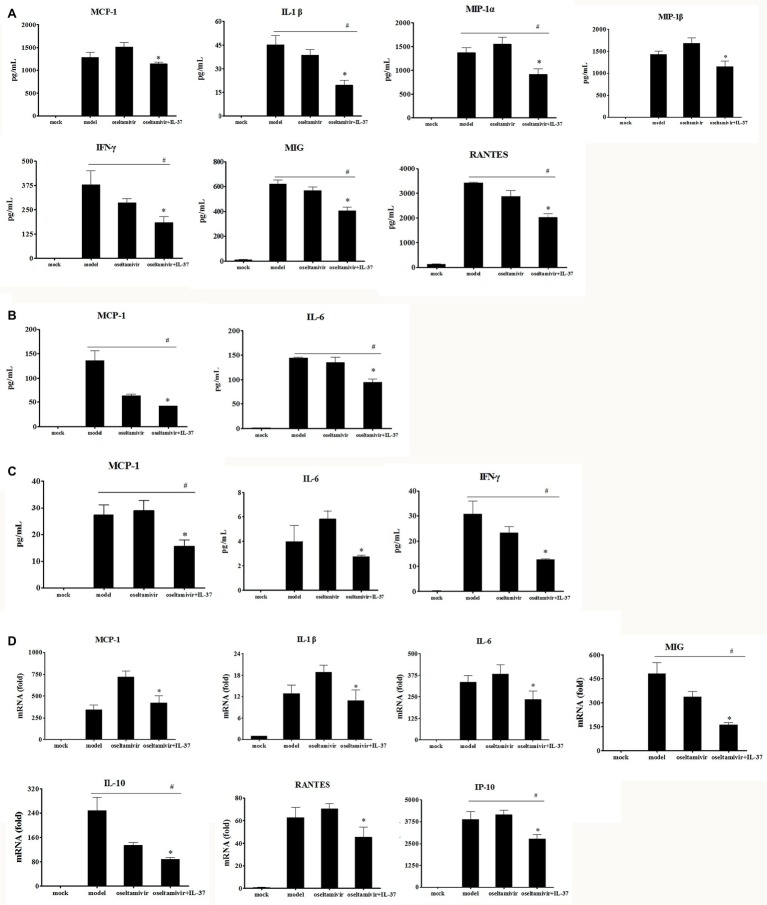
IL-37 treatment modulates immune responses to H1N1 infection. BALF, serum and lung homogenates were harvested 6 d.p.i. following IL-37 treatment. The levels of G-CSF, GM-CSF, IFN-γ, MIG, IL-1α, IL-1β, IL-4, IL-5, IL-6, IL-10, IL-12p70, IL-13, IL-17A, RANTES, MCP-1, MIP-1α, MIP-1β, and TNF, in lung homogenates **(A)**, BAL fluid **(B)** as well as serum samples **(C)** were measured quantitatively using a Cytometric Beads Array. There were no significant differences in the concentrations of other cytokines and chemokines tested (data not shown). **(D)** The relative mRNA expression of cytokines in lung tissues were detected by real-time RT-PCR. Data are representative of three independent experiments with three mice for each group. *Significant difference (*p* < 0.05), compared with oseltamivir-inoculated mice. ^#^ Significant difference (*p* < 0.05), compared with H1N1-infected mice.

IL-37, as a potent inhibitor of innate immunity, can shift the cytokine equilibrium away from excessive inflammation (Teng et al., 2014). Thus, to more accurately assess the efficacy of IL-37 during H1N1 infection, the mRNA expression and the protein production levels of IL-6, TNF-α, MCP-1, IL-1α, IL-1β, MIP-1α, MIP-1β, IP-10, MIG, RANTES, IFN-γ and IL-10 were detected by RT-PCR and CBA in the lung tissue, BALF and serum samples on day 6 after H1N1 infection. As expected, IL-37 treatment inhibited the increase in levels of MCP-1, IL-1β, MIP-1α, MIP-1β, MIG, IFN-γ and RANTES in the lungs of the model group (Figure 4A). Paralleling the decreased production of cytokines, the upregulation of MCP-1, IL-1β, IL-6, IP-10, MIG, and RANTES mRNA expression was markedly reduced in the IL-37 treatment group (Figure 4D). In addition, it is worth mentioning that the expression of MCP-1 and IL-1β, especially the level of MCP-1 in the oseltamivir group, was higher than that in the model group; however, oseltamivir plus IL-37 treatment corrected the increase in MCP-1 expression (Figure 4D). Moreover, the mRNA expression of the anti-inflammatory cytokine IL-10 was downregulated in the IL-37-treated group (Figure 4D); however, the production of IL-10 protein in the lungs was not significantly changed (data not shown).

Furthermore, compared with that in the model and oseltamivir groups, the upregulated production of MCP-1, IL-6 and IFN-γ in the BALF (Figure 4B) and serum (Figure 4C) was markedly reduced in the IL-37 treatment group. These results indicate that IL-37 exerts a protective effect by regulating the levels of inflammatory cytokines, particularly by regulating macrophage cytokine production.

### Macrophage Percentages Are Increased in Interleukin-37 Treated Mice

**Figure 5:**
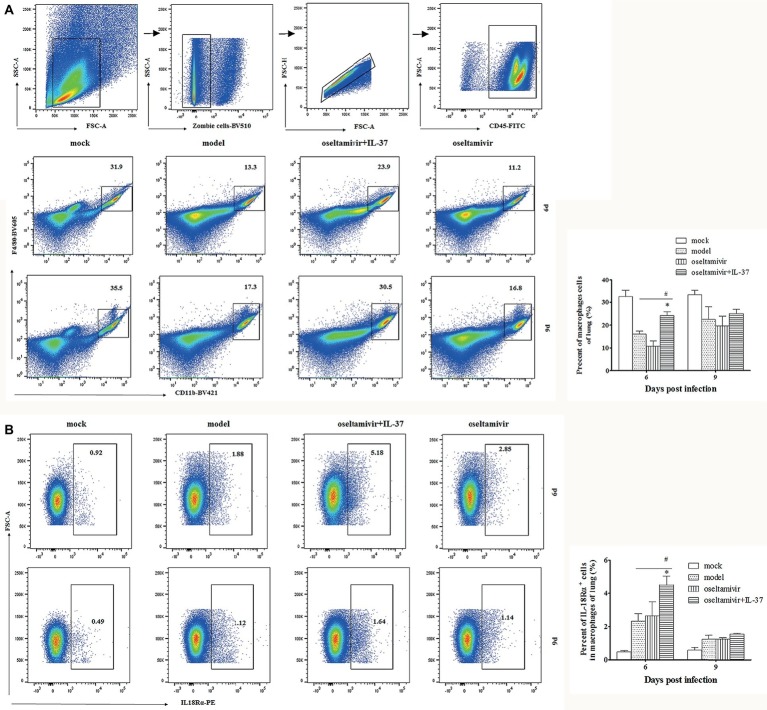
The macrophage percentages are increased in IL-37-treated mice. The percentage of CD45^+^F4/80^+^CD11b^+^ macrophages **(A)** and IL-18Rα^+^ macrophages **(B)** in the lungs of mice was determined by flow cytometry at the indicated time points. Data are representative of three independent experiments with three mice for each group. ^*^Significant difference (*p* < 0.05), compared with oseltamivir-treated mice. ^#^Significant difference (*p* < 0.05), compared with H1N1-infected mice.

To evaluate the roles of immune cells in the IL-37-mediated protection against influenza A (H1N1) infection, the changes in the numbers of macrophages and T cells in the lungs of different groups at multiple time points were analyzed using flow cytometry. In fact, the percentages of CD4^+^ and CD8^+^, IL-18Rα^+^CD4^+^ or CD8^+^ T cells in the IL-37 treatment group were not obviously different compared with those in the model group (Supplementary Figure S1). However, an increase in the percentage of macrophages, which were identified as CD45^+^F4/80^+^CD11b^+^ cells, was observed in the lungs of mice in the IL-37 administration group, peaking at day 6 post infection (Figure 5A). Paralleling the augmented macrophage percentage, the percentage of IL-18Rα^+^ macrophages was markedly enhanced in the lungs of IL-37-treated mice (Figure 5B). These results show that IL-37 administration impairs the decrease in the macrophage population in the lungs, indicating that macrophages may exert immunoprotective effects in mice treated with IL-37 during H1N1 infection.

### Depleting Macrophages Reduces the Protective Effect of Interleukin-37 During Influenza Virus Infection

**Figure 6:**
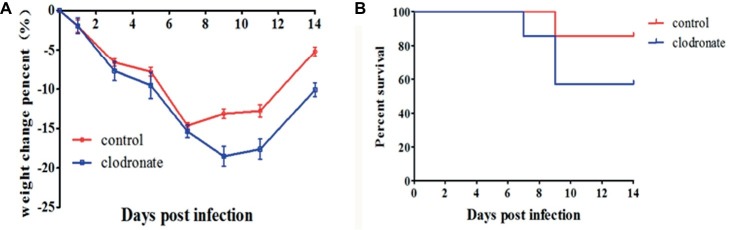
Macrophage depletion reduces the protective effect of IL-37 during influenza virus infection. Body weights **(A)** and survival rates of **(B)** mice treated with clodronate liposomes or control liposomes during IL-37 administration. Data are presented as the average values from two independent experiments ± SD (*n* = 7 per group).

As macrophages may exert immunoprotective effects in H1N1-infected mice treated with IL-37, we intranasally administered clodronate liposomes, which have been shown to deplete macrophages in the lungs (Tate et al., 2010; Wong and Smith, 2017), to mice during influenza virus infection to test the dependency of the IL-37 treatment-induced reduction in mortality on macrophages. As expected, under the same conditions, the clodronate liposome administration group reached its lowest weight at 9 d.p.i. and the body weight change did not recover in the control liposome group (Figure 6A). Moreover, clodronate liposome treatment significantly increased the mortality rate (Figure 6B). These data further demonstrate that macrophages are critical for the protective effect of IL-37 during influenza virus infection.

### Interleukin-37 Inhibits the Expression of Inflammatory Cytokines in a MAPK-Dependent Manner *in vitro*

**Figure 7:**
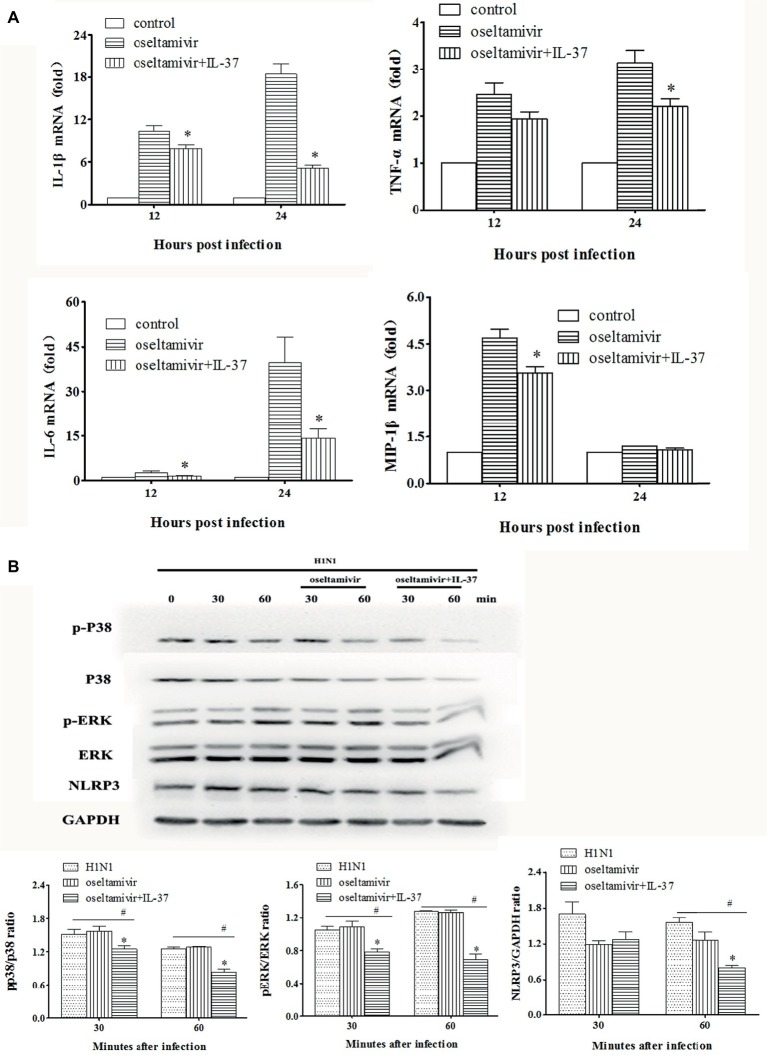
IL-37 inhibited the expression of inflammatory cytokines in MAPK-dependent pathway *in vitro*. **(A)** Effects of IL-37 on the mRNA expression of MCP-1, IL-6, MIP-1α, MIP-1β, TNF-α, etc. in RAW264.7 cells on 12, and 24 h after infection. **(B)** Representative photographs of western blot in protein expressions of the phosphorylated and non-phosphorylated forms of ERK1/2, P38 MAPK, NLRP3 and GAPDH in RAW264.7 cells with or without IL-37 treatment during H1N1 infection. The data are representative of three independent experiments. ^*^Significant difference (*p* < 0.05), compared with oseltamivir-inoculated mice. ^#^Significant difference (*p* < 0.05), compared with H1N1-infected cells.

To clearly demonstrate the anti-inflammatory efficacy of IL-37, following H1N1 infection, murine macrophage RAW264.7 cells were treated with IL-37 for different times (12 and 24 h). The levels of inflammatory factors in the cells were determined by real-time PCR. As shown in Figure 7A, IL-6 mRNA expression at all time points was obviously downregulated in the IL-37 treatment groups compared with the oseltamivir group. Similar inhibitory effects were also observed on TNF-α, IL-1β and MIP-1β expression (Figure 7A).

To further explore the underlying mechanisms of IL-37, we examined the expression of PRR-related protein phosphorylation. For this purpose, western blot analyses were performed using the cell lysates of RAW264.7 cells treated with or without IL-37 to analyze the phosphorylation of MAPKs and GAPDH. In addition, NLRP3 protein production was detected. As shown in Figure 7B, compared with that in the infection and oseltamivir groups, the phosphorylation of ERK1/2 and p38 MAPK was significantly reduced in the IL-37 treated RAW264.7 cell group. Similarly, the ratio of NLRP3 in the IL-37-treated group was significantly decreased *in vitro*. These results show that IL-37 treatment inhibits the production of macrophage inflammatory cytokines induced by H1N1 infection in a MAPK-dependent manner.

## Discussion

Influenza viruses cause seasonal epidemics and sporadic pandemics, and are a major burden on human health. The rapid development of viral pneumonitis induced by aggressive inflammation resulting in high morbidity and mortality, emphasizing the importance of exploring effective approaches to ameliorate the viral pneumonia during H1N1 infection (Morita et al., 2013). IL-37 has been shown to block the deleterious effects of pro-inflammatory stimuli or conditions in numerous models (Barbier et al., 2019; Theoharides et al., 2019), it is essential for the inhibition of innate immunity and inflammation and plays a role in the inhibition of cytokine and chemokine production, and inflammatory cell infiltration (Theoharides et al., 2019). Increasing evidence suggests that the anti-inflammatory cytokine IL-37, improve functional outcomes in combination, including neuroprotection and reduced of lung infection burden (Zhang et al., 2019).

In the present study, by using H1N1-infected BALB/c mice, we found that intravenous IL-37 treatment advanced the time to body weight recovery onset, improved the survival rate (Figures 1, 2), and ameliorated the increase in the exudation of inflammatory proteins in the alveoli (Figure 3). These results demonstrate that IL-37 treatment can ameliorate viral pneumonia and afford a better protection from A/California/07/2009 (H1N1) infection in the murine model. Furthermore, IL-37 treatment significantly reduced the production of the inflammatory cytokines and chemokines MCP-1, IL-1β, MIP-1, IFN-γ, MIG and RANTES, meanwhile the increased mRNA expression of MCP-1, IL-1β, IP-10, IL-10, MIG and RANTES in the lungs of the IL-37-treatment group was obviously decreased compared with that in the oseltamivir group (Figure 4). Interestingly, most of the cytokines obviously decreased at both the transcriptional and translational levels were macrophage cytokines.

Indeed, IL-37 administration impaired the decrease in the percentage of macrophages in the lungs of H1N1-infected mice (Figure 5), and depleting macrophages reduced the protective effect of IL-37 during influenza virus infection (Figure 6). The anti-inflammatory endogenous ligand annexin A1 has been shown to attenuate pathology upon subsequent influenza A virus infection, and reduction in lung damage severity is associated with an increase in the number of alveolar macrophages (AMs) in the murine model of influenza A virus infection (Schloer et al., 2019). Numerous literatures have demonstrated that macrophage are critical for host defense in mice during influenza viral infection (He et al., 2017; Wong and Smith, 2017). Our results are consistent with the results of these reports, showing that macrophages may exert immune protective effects in H1N1-infected mice treated with IL-37.

IL-37 can strongly regulate macrophages to restrain the autoimmune response (Ye and Huang, 2015; Toulmin et al., 2017; Wang et al., 2018; Yang et al., 2019). It has been reported that IL-37 can promote macrophage polarization from the pro-inflammatory subtype (M1) to the anti-inflammatory subtype (M2) in atherosclerosis (McCurdy and Baumer, 2017). Moreover, IL-37 induces a phenotypic shift in THP1-derived macrophages toward a CD206^+high^ and CD86^+low^ macrophage subtype and enhanced the mRNA levels of IL-10, which are characteristic hallmarks of M2 macrophages (Huang et al., 2015). In summary, these results indicate that IL-37 treatment ameliorates the lung damage by polarizing macrophages from an M1 to an M2 phenotype. Further research regarding the mechanisms of the inhibitory effect of IL-37 is needed.

To further confirm that macrophages play an important role in the anti-inflammatory effect of IL-37, RAW264.7 cells were infected with H1N1, and treated with oseltamivir in combination with/without IL-37. At the indicated intervals, macrophages were collected, and the expression of cytokine mRNA in the cells was detected. As shown in Figure 7A, compared with that in the oseltamivir group, the mRNA expression of IL-1β, IL-6, TNF-α and MIP-1 was obviously downregulated in the IL-37 treatment group. These results are consistent with those of pulmonary studies, which further indicates that IL-37 treatment can ameliorate H1N1-induced inflammation by reducing macrophage cytokine production.

The activity of IL-37 has been reported to largely depend upon IL-18Rα and SIGIRR for the extracellular activation of the anti-inflammatory pathway (Lunding et al., 2015; Zeng et al., 2017). Herein, we found that paralleling the augmented macrophages percentage, the IL-18Rα^+^-macrophages percentage was enhanced markedly in the lungs of IL-37-treated mice (Figure 5), indicating that IL-37 down-regulates the increased production of pro-inflammatory cytokines in an IL-18Rα activation-dependent manner. Then, by using the RAW264.7 cell line, the underlying mechanisms of the IL-37 effect in macrophages were further investigated. Studies have shown that MAPK-related signaling can be inhibited by IL-37 in activated mast cells (Gallenga et al., 2019). In addition, the intraperitoneal injection of IL-37 significantly decreases the expression of NLRP3 in the mouse lung aspergillosis model (Moretti et al., 2014; Jia and Liu, 2018). Indeed, our results showed that the increased phosphorylation of ERK1/2 and p38 MAPK was significantly downregulated in RAW264.7 cells treated with IL-37; furthermore, the ratio of NLRP3 in the IL-37-treated group was decreased *in vitro* (Figure 7). In contrast, treatment with IL-37 did not inhibit the production of JNK protein in influenza A virus-infected RAW 264.7 cells (data not shown). These results confirm that IL-37 ameliorates influenza pneumonia by attenuating macrophage cytokine production in a MAPK pathway-dependent manner, especially the ERK1/2 and p38 pathways.

In conclusion, these data provide evidence that IL-37 inhibits the pathogenesis of influenza pneumonia by decreasing the production of essential pro-inflammatory cytokines, indicating a new and promising therapeutic approach for excessively activated immune responses in influenza A infection-induced pneumonia. However, the function of IL-37 in other viral infections, especially serious emergent and re-emerged infectious diseases, remains unclear. Further detailed research remains necessary to fully determine the possible functions of IL-37 in viral infections.

## Data Availability Statement

All datasets generated for this study are included in the article/Supplementary Material.

## Ethics Statement

The animal study was reviewed and approved by The Institute of Animal Use and Care Committee of the Institute of Laboratory Animal Science, Peking Union Medical College.

## Author Contributions

CQ, LB, and FQ conceived, designed, and supervised the experiments. FQ and ML performed most experiments, analyzed the data, and wrote the original draft. LB, FQ, and ML analyzed the data. FL, QL, GW, SG, SW, and YX conducted some experiments. FQ and ML edited the manuscript. All authors reviewed and approved the manuscript.

### Conflict of Interest

The authors declare that the research was conducted in the absence of any commercial or financial relationships that could be construed as a potential conflict of interest.

## Supplementary Material

SUPPLEMENTARY FIGURE S1 Percentages of lymphocytes were detected in IL-37-treated mice. **(A)** The percentages of different lymphocytes that were identified as CD3^+^CD4^+^ or CD3^+^CD8^+^ as well as IL-18Rα^+^ lymphocytes in the lungs of IL-37 treated mice were determined by flow cytometry on day 6 during H1N1 infection. Data are representative of three independent experiments with three mice for each group. ^*^Significant difference (*p* < 0.05), compared with oseltamivir-treated mice.
