# Supplementary figures and images for: Interleukin-37 Ameliorates Influenza Pneumonia by Attenuating Macrophage Cytokine Production in a MAPK-Dependent Manner
Source: Front Microbiol. 2019 Oct 30;10:2482. doi: 10.3389/fmicb.2019.02482 (PMC6831648; doi:10.3389/fmicb.2019.02482)

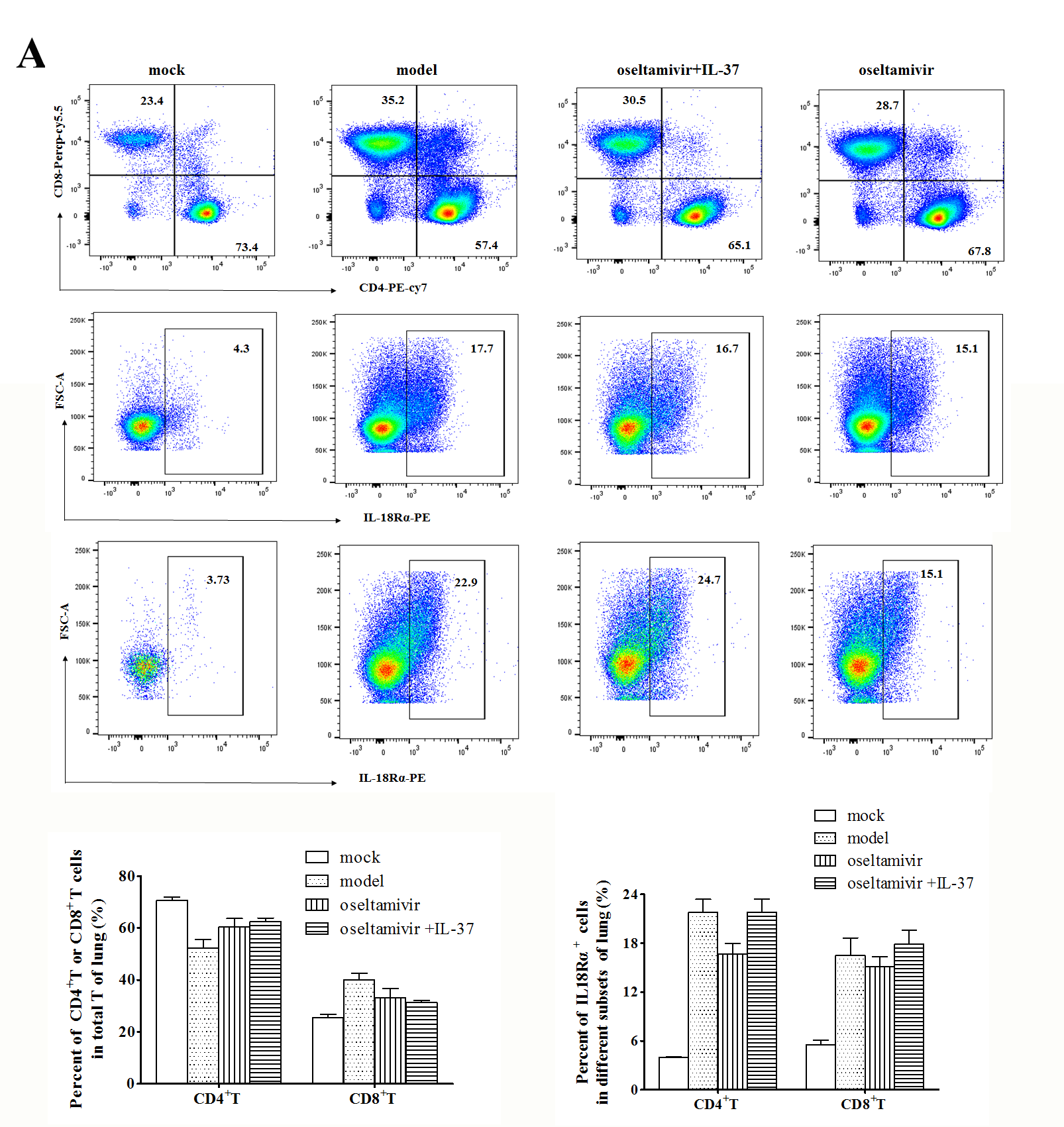

Supplement: SUPPLEMENTARY FIGURE S1 — Percentages of lymphocytes were detected in IL-37-treated mice. (A) The percentages of different lymphocytes that were identified as CD3+CD4+ or CD3+CD8+ as well as IL-18Rα+ lymphocytes in the lungs of IL-37 treated mice were determined by flow cytometry on day 6 during H1N1 infection. Data are representative of three independent experiments with three mice for each group. *Significant difference (p < 0.05), compared with oseltamivir-treated mice. [file Image_1.TIF]
